# Design, Development and FE Thermal Analysis of a Radially Grooved Brake Disc Developed through Direct Metal Laser Sintering

**DOI:** 10.3390/ma11071211

**Published:** 2018-07-13

**Authors:** Gulam Mohammed Sayeed Ahmed, Salem Algarni

**Affiliations:** Department of Mechanical Engineering, College of Engineering, King Khalid University, P.O. Box 394, Abha 61421, Saudi Arabia; saalgarni@kku.edu.sa

**Keywords:** design, disc brake, 3D metal printing, direct metal laser sintering, thermal stress analysis, radial grooves

## Abstract

The present research work analyzed the effect of design modification with radial grooves on disc brake performance and its thermal behavior by using additive manufacturing based 3D printed material maraging steel. Temperature distribution across the disc surface was estimated with different boundary conditions such as rotor speed, braking pressure, and braking time. Design modification and number of radial grooves were decided based on existing dimensions. Radial grooves were incorporated on disc surface through Direct Metal Laser Sintering (DMLS) process to increase surface area for maximum heat dissipation and reduce the stresses induced during braking process. The radial grooves act as a cooling channels which provides an effective means of cooling the disc surface which is under severe condition of sudden fall and rise of temperatures during running conditions. ANSYS software is used for transient structural and thermal analysis to investigate the variations in temperatures profile across the disc with induced heat flux. FE based thermo-structural analysis was done to determine thermal strains induced in disc due to sudden temperature fluctuations. The maximum temperature and Von Mises stress in disc brake without grooves on disc surface were observed which can severely affect thermal fatigue and rupture brake disc surface. It was been observed by incorporating the radial grooves that the disc brake surface is thermally stable. Experimental results are in good agreement with FE thermal analysis. DMLS provides easy fabrication of disc brake with radial grooves and enhancement of disc brake performance at higher speeds and temperatures. Therefore, DMLS provides an effective means of implementing product development technology.

## 1. Introduction

**Figure 1:**
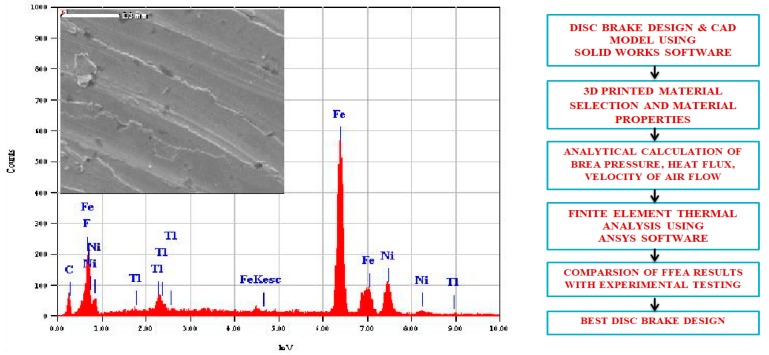
3D printed maraging steel Characterization, flow chart of Design-Analysis of disc brake.

**Figure 2:**
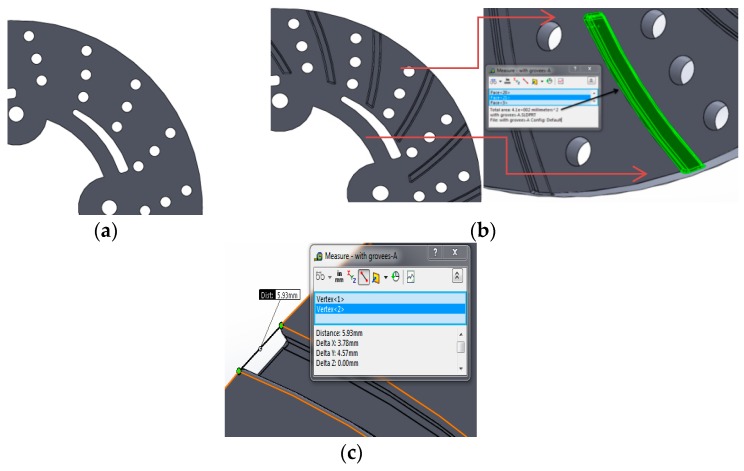
3D-CAD geometries: (**a**) without Radial grooves; (**b**) with radial grooves; and (**c**) optimal dimensions and areas of grooves.

One of the remarkable additive manufacturing (AM) processes that allows direct production by means of material layer addition is direct metal laser sintering, which was developed to overcome the disadvantages of traditional processes. DMLS process allows the direct production of components at net shape quality. The major advantage of AM process is fewer post-production processes, no geometrical restrictions, and a smallest possible feature size of about 100 µm. DMLS uses laser based processing techniques with different variety of 3D printed materials such as polymers, metals, ceramics and composites. The main objective of this research work was to fill the observed research gap on avoiding undesirable thermal strains, thermal fatigue and thermal damage on disc surface due to sudden variations in thermal boundary conditions. This research work designed a disc brake with radial grooves on disc surface. These modifications are achieved by using additive manufacturing based 3D printed DMLS processes in fabrication. Murr et al. [1] used a DMLS machine equipped with powder feed, bed, and laser energy sources for sintering and melting. A CAD model of the energy provided to metal powder during DMLS is based on full melting of powdered state of material by a high power laser beam (100–500 W). The starting 3D printed material in the form of powder is deposited on a substrate plate; the deposited layer is some tens of microns (25–50 µm). Different types of metals can be processed through DMLS; most common metals used for this process are aluminum alloys, nickel alloys, tool steels and stainless steels. In this present work, maraging steel was considered to study thermal behavior of 3D printed metal under sudden increase and decrease of temperature conditions. Maraging steel evolves from a group of martensitic steels with less carbon content. These steels are designated from the combination of “martensitic” and “aging”, since these steels go through different aging heat treatments to improve strength and hardness. Maraging steel is most suitable for DMLS, since they have good weldability property at micro level due to micron-sized melt-pool in the DMLS process with high cooling rates. The property of these steels is proven to be well-matched with typical heavy duty applications in aerospace, automobile and tooling industries. The most commercially available grades for maraging are 200, 250, 300 and 350, which specify yield stress in kilo-pound per square inch with nominal yield stress for maraging being from 1500 to 2500 MPa. Vickers Hardness value in 10 kgf is HV123 (10 Kg), density is 8.2082 g/cc, temperatures range is from 450 to 650 °C for ageing treatment and proper selection of working temperatures must be made for desirable strength for reasonable time interval. Maraging steels are applied for applications with less cobalt and nickel content and have been produced to decrease costs. The strength of Martensite is controlled by carbon content, while Ni and Al have less effect than other alloying elements. Electron dispersive X-ray spectroscopy (EDX) reveals 3D printed Maraging Steel (MS1) characterization, as shown in Figure 1. Each element has unique emission spectrum. Measuring the spectrum peak intensities after an appropriate calibration, a quantitative estimation of chemical composition can be attained for material composition of maraging steel given in Table 1. The requirement for metallic 3D printing is expected to grow faster than plastic. In the present context, a research gap has been observed to avoid these undesirable thermal strains, thermal fatigue, and thermal damage on disc surface due to sudden variations in thermal boundary conditions. Thus, this research work designed a disc brake with modifications on disc surface with radial grooves and these modifications were achieved using DMLS processes for easy fabrication. Today, automotive manufacturers use the 3D printing technology for prototyping and functional parts manufacturing. In Figure 2, 3D-CAD geometries are shown without and with radial grooves having optimal dimensions of grooves. 3D printing can also improve quality through lighter parts, better ergonomics and more design freedom. Automotive companies also found overall product cycle times decreases by experimenting with 3D printing for assembly fixture, customized fixtures and tooling, making parts cheaper, lighter and faster is often a key goal of automotive industry, indicating opportunities for 3D printing manufacturing.

## 2. Development of Disc Brake by DMLS

DMLS based additive manufacturing process refers to digital 3D CAD data being used to build up a solid model in layers by depositing molten metal, and helps in developing complex products that can be light and stable [2]. The solid model during laser sintering is prepared by positioning and slicing through 3D printing software, namely Magics RP. The STL format of CAD data is converted into layer data by means of buildup processor available in 3DP machine. DMLS machine has building volume of 250 mm × 250 mm × 325 mm, equipped with fiber laser of 400 W having scanning speed of 7 m/s [3]. During DMLS, metal powder upon exposure to laser power beam melt droplets are created and, due to moving beam, melt pools are created and can be regarded as small castings. 3D printed maraging steel properties are given in Table 2. After getting final 3D printed disc brake of maraging steel, it was heat treated for enhancing hardness and mechanical properties, dimensional stability, reduction in residual stresses, corrosion cracking, and fatigue. Ageing temperature is 490 °C for 5–8 h with air cooling. The 3D printing machine EOS M-290 is a flexible, fast and cost-effective production system for metallic parts. In this work, 3D printed maraging steel (MS1) was considered due to its remarkable properties, such as excellent strength, high toughness, excellent surface finish, and good thermal conductivity. Herzog et al. [4] showed that maraging steel provides applications in critical parts in automotive, aerospace, structural component, tooling, machine tools, fasteners and production sectors. 

**Figure 3:**
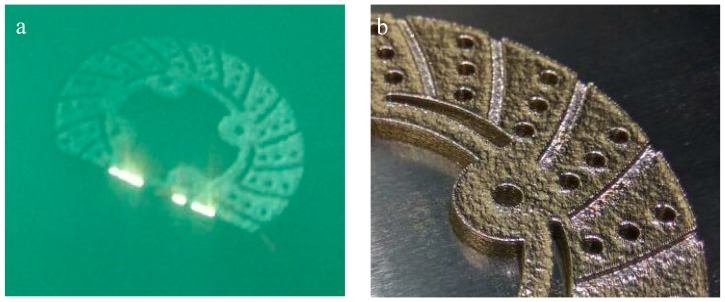
(**a**) Development of brake disc in EOS M-290 and (**b**) Disc brake developed by DMLS.

**Figure 4:**
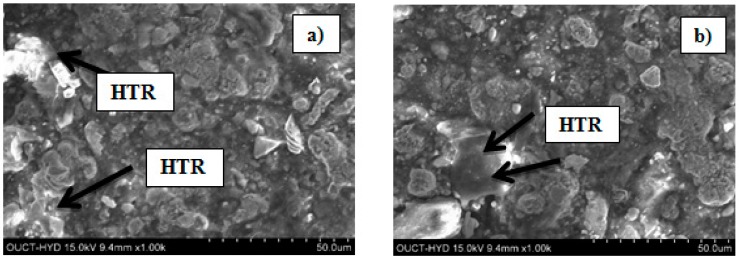
SEM of brake disc surface subjected to High Temperature Region (HTR) at: (**a**) R = 50 mm; and (**b**) R = 100 mm.

**Figure 5:**
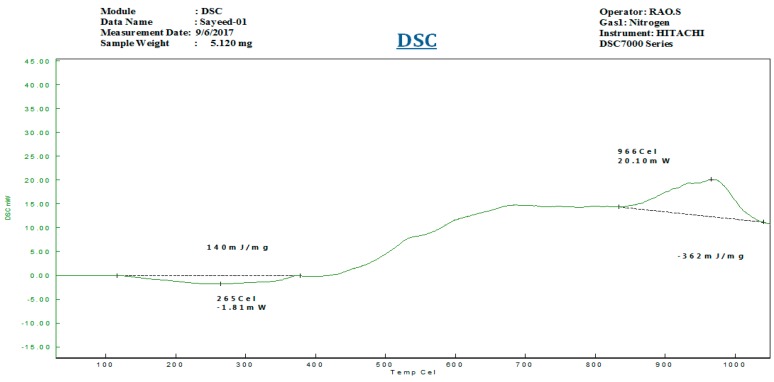
Differential Scanning (DSC) analyses of maraging steel from ambient to 1000 °C.

The of brake disc developed by EOS M-290 system and disc brake developed by DMLS process are depicted in Figure 3. Micrographs of disc brake surface, powder morphology analysis of top surfaces of disc brake under heat flux and temperature surface conditions were carried out by Zeiss EVO 50 (ZEISS, Jena, Vienna, Germany) scanning electron microscope (SEM). SEM micrograph of brake disc surface, shown in Figure 4 at radii 50 mm and 100 mm, represents the high temperature region of maraging steel by means of high energy collimated electron beam Leitz Aristomet (Leitz, Hicksville, NY, USA) microscope with high resolution and high depth of focus. Differential scanning calorimetry analyses, shown in Figure 5, were performed using HITACHI (Hitachi Systems, Chiyoda, Tokyo, Japan), Differential Scanning Calorimeter DSC7000 Series, TGDSC-DTA equipment at KELVIN LABS (Kelvin Lab Inc, Hyderabad, Telengana, India), in nitrogen gas atmosphere at three different heating rates (30, 40, and 50 °C/min) between 30 and 1000 °C, with scanning rate 0.01 °C to 150 °C/min, TG measurement range as ±400 mg. Maraging specimens were prepared by cutting small samples having a weight of about 5.120 mg. DSC tests were conducted to assess phase changes and reactions sequence. The DSC testing instrument consists of empty crucible and another one containing the maraging specimen. They are simultaneously heated and kept at the certain temperature. A DSC curve reveals heat flow, i.e. amount of energy exchanged by specimen versus subjected temperature. DSC makes it possible to study phase transformation sequence under precise non-isothermal temperatures. It was observed in DSC curve that MS1 specimen is stable. No phase change was observed during the testing temperature between 300 and 1000 °C. Typical DSC Specifications are given in Table 3.

## 3. Previous Studies on Thermal Analysis of Disc Brake

Belhocine et al. [5] presented a simulated thermal behavior in brake disc and determined the initial flux entering the disc to evaluate convective coefficient and visualize disc temperature (3D). We conclude that temperature is influenced by construction and materials and mode. Choi et al. [6] reported that sudden rise and fall of temperature change in metal parts of sliding systems induces uneven thermal stresses due to thermal expansion. This phenomenon is particularly evident in disc brakes under high thermal loads. This paper deals with the finite element modeling of frictional heating process in disc brakes to study the temperature and stress distributions during operation. Andinet et al. [7] presented factors influencing braking performance of train during braking time and found major factors are temperatures and friction coefficient between pad and brake disc. Thermal transient analysis of disc braking system was performed to evaluate nodal temperature under different thermal and operating conditions. Balaji et al. [8] reported thermal degradation is significant to determine thermal stability of product considering the brake application. The present paper deals with the role of various fibers such as aramid, acrylic and cellulose fibers. Thermogravimetric analysis has shown that composite NA03 had minimum weight loss and more thermal stable. Grzes et al. [9] and Marko et al. [10] reported the influence of the pad cover angle on temperature fields on disc brake. A three-dimensional finite element (FE) model of pad–disc system was developed and calculations were carried out for single braking process at constant deceleration with contact pressure corresponds with cover angle of the pad and the evaluated distributions of temperature for both contact surface of pad and disc surface. They developed and evaluated three-dimensional (3D) thermal–structure coupling model and implemented transient thermal analysis of thermo-elastic contact of disc brakes with variation in frictionally generated heat. They found the source of thermal fatigue was the thermo-elastic problem using finite element method. The results demonstrate that the maximum surface equivalent stress may exceed the material yield strength during an emergency braking, which may cause a plastic damage accumulation in a brake disc, while residual tensile hoop stress is incurred on cooling. FE structural and thermal analysis has been carried out with the dimensions and specifications of Corolla car model. The demand of this automobile car is increasing worldwide due to its low cost, high fuel economy and different towing capacity as per the requirement of roads. The maximum speed of this car is 200 kph. This high speed is the reason this car was considered for structural–thermal analysis of disc brake, and maximum speed was used for thermal analysis. Dimensions of disc and pads given in Table 4 were used to develop the 3D model in solid works with and without radial grooves. The average stopping distance with fully loaded disc brake at 25 °C traveling speed varies from 100 to 200 m under the experimental test conditions, requiring an average of 81 m stopping distance with the deceleration rate 8 m/s^2^ in 4.5 s. For the analysis, speed of the car reduced from 33.34 to 0 m/s, within 4.5 s. Single stop cycle of braking was used for thermal and structural analysis since material regains its original elastic condition after brake force is removed. Lopez et al. [11] made several assumptions to simplify thermal analysis complexity and output surface temperature was measured experimentally and compared with FE analysis [12,13]. Heat dissipated through brake disc surface during application of brake and heat flux applied to surface was considered with and without radial grooves. Huajiang et al. [14] considered heat transfer convection only after brake application was completed and car accelerated to regain its original speed. These areas include an effective surface area for applying braking pressure with and without radial grooves on disc surface. The remaining surface area of the disc was considered insulated for the purpose of comparison of surface temperatures with and without grooves under the brake pressure of 1 MPa.

## 4. Temperature Distributions in Disc Brake

To investigate temperature behavior of brake discs, it is necessary to obtain temperature distributions as a function of braking time, speed, and braking pressure. This research attempted to incorporate radial groove features on disc surface by using DMLS and to predict the temperature response due to these design modifications. Rotary motion of the brake disc causes the sliding surface contact between pad and disc to generates heat. Hudson et al. [15] showed that the surface temperature due to friction generated heat should be considered. The friction force is determined from pressure distribution at contact surfaces of disc and pad. According to Mahmoud et al. [16], the kinetic energy of a car, once the brakes are applied to the pads, which press against brake rotor, is converted to thermal energy [17]. In the case of disc brakes, it is kinetic energy converted to thermal energy. $M_{v}$ is total mass of vehicle and $V_{i}$ is initial speed of vehicle and the heat dissipated by each disc is Ke=12MvVi2, i.e., the rate of heat generated due to friction is equal to friction power, and this frictional heat is absorbed by brake disc and pads. If it is assumed that whole friction power is transferred to heat energy, then heat partition $\gamma_{p}$ coefficient needs to be considered. Thermal energy generated at friction surface of brake can be transferred to both rotor and pads. This partitioning of energy is dependent on thermal resistances of brake discs and pad that are further dependent on density, material thermal conductivities and heat capacities. The brake pad thermal resistance must be more than rotor thermal resistance to avoid brake fluid from high temperatures. The partitioning coefficient (γ) for thermal input to brake disc and pad was determined from thermal effusivity ξ given by

(1) $$\xi_{\mathrm{ed}}=\sqrt{k_{d}\rho_{d}c_{d}},\xi_{\mathrm{ep}}=\sqrt{k_{p}\rho_{p}c_{p}}$$

The friction contact area of pad and disc are determined from equations given as

(2) $$A_{\mathrm{cp}}=\emptyset_{c}\int_{r_{1}}^{r_{2}}\mathrm{r dr},A_{\mathrm{cd}}=2\times \pi \int_{R1}^{R_{2}}\mathrm{R dr}$$

**Figure 6:**
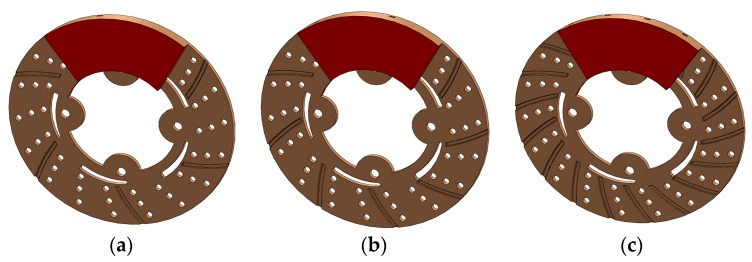
CAD model of brake disc and pad with (**a**) 6, (**b**) 9 and (**c**) 18 radial grooves on disc surface.

The total heat generated on frictional contact interface $Q_{\mathrm{Total}}$ equals the heat flux into the disc $Q_{\mathrm{disc}}$ and pad $Q_{\mathrm{Pad}}$, and braking energy, which is termed as heat partition coefficient $\gamma_{p}$, is determined from the following equation:

(3) $$\gamma_{p}=\frac{\xi_{\mathrm{ed}}A_{\mathrm{cd}}}{\xi_{\mathrm{ed}}A_{\mathrm{cd}}+\xi_{\mathrm{ep}}A_{\mathrm{cp}}}$$

where $\xi_{\mathrm{ed}}$ and $\xi_{\mathrm{ep}}$ are thermal effusivities of disc and pad; this partitioning of thermal energy is dependent on thermal resistances of pad and brake disc rotor, which are related to their thermal conductivities, materials densities, and heat capacities. Solid works CAD models of disc and pad with 6, 9 and 18 radial grooves on disc surface are shown in Figure 6. The heat flux generated by pressing pad against rubbing surface of rotor is only source of heat input to the disc; magnitude of this heat flux was calculated from basic energy principle and input of energy is in terms of rotor disc speed, radius of rotor, coefficient of friction and pressure distribution [18]. The frictional heat generation due to friction of contact surfaces of two surfaces of brake system, coefficient of friction, speed of vehicle, geometry of the disc rotor and pad, and pressure distribution at the sliding surfaces. In uniform pressure distribution, heat flux $Q_{\mathrm{Total}}$ on contact area under the pressure distribution is taken into account in thermal and structural analysis [19].

In the present thermal analysis, ambient temperature was assumed at 25 °C and brake disc surface temperature was 37.2 °C at 100 kph. It was assumed that heat dissipation from brake disc surface to atmosphere through convection process is governed by $Q_{f}=h_{C}A_{\mathrm{Cd}}\left(T_{s}-T_{a}\right)$, where Q_f_ is in Watts; hc is the convection heat transfer coefficient; Input parameters and dimensions of brake discs are tabulated in Table 5, Acd and Acp are the contact surface area of the disc and pads, respectively, in m^2^; Ts is surface temperature of brake disc; and Ta is ambient air temperature in °C. Heat transfer coefficient is applied to brake discs as heat flux boundary condition. Thus, increasing the rate of heat transfer from surface brake discs reduces disc surface temperature on total surface area of the brake discs [20].

Density of air $\rho_{\mathrm{air}}$ (kg/mm^3^) is given by $=1.225\;\mathrm{Kg}/m^{3}$, where $m_{a}$ is the mass flow rate of air (m^3^/s) and $V_{\mathrm{avge}}\;$ is the average air velocity (m/s). Convective heat transfer coefficient at different air velocities are obtained from the formula [21].

(4) $$h_{c}=0.70{\;K}_{\mathrm{air}}/d_{o}{\left(R_{e}\right)}^{0.55}$$

(5) $$V_{\mathrm{avge}}=0.015\omega \left[\frac{A_{\mathrm{outet}}+A_{\mathrm{inlet}}}{A_{\mathrm{outet}}}\right]{\left[d_{o}^{2}-d_{i}^{2}\right]}^{0.5}m/\sec$$

Radial grooves areas at inlet and outlet are $8.75{\;\mathrm{mm}}^{2}$ Air thermal conductivity $K_{\mathrm{air}}=0.024\;W/\mathrm{mK}$, $\mathrm{The}\;\mathrm{contac}\;\mathrm{areas}\;\mathrm{of}\;\mathrm{the}\;\mathrm{pad}\;\mathrm{and}\;\mathrm{disc}\;\mathrm{are}\;0.0061236{\;m}^{2},\;0.033912{\;m}^{2}$, respectively. The Convective heat transfer $Q_{f}$ into disc surface can be calculated using Equation (6) [22].

(6) $$Q_{f}\;=\;\frac{1-\emptyset }{2}\frac{{\mathrm{gmzV}}_{0}}{2A_{\mathrm{cd}}\varepsilon_{p}}$$

where Ø is rate coverage sector of braking forces between the front and rear axle, Z=adg is braking effectiveness, A_cd_ is disc surface swept by brake pad (m^2^), ε_p_ is factor load distributed on brake disc surface, m is mass of vehicle (kg), g = 9.81 is acceleration of gravity (m/s^2^), V_0_ is initial speed of vehicle (m/s), and ad is the deceleration of the vehicle (m/s^2^).The disc brake groove passage and sector chosen for numerical analysis are shown in Figure 1. The dimensions of grooves on disc surface are 3.5 mm by 2.5 mm each, with outer and inner diameters as 240 mm and 120 mm, respectively. Heat transfer coefficient $h_{C}$ associated with laminar flow for radial and non-radial grooves on brake discs was calculated for Re < 2.4 × 10^5^, where do is outer diameter of discs mm, Re is Reynolds number, and Ka is thermal conductivity of air, W/m °C. Experimental validation was done on modified brake disc with and without radial grooves brake using non-contact thermometer Fluke-561, Infrared thermometer, which can measure contact and ambient temperatures. IR thermometer is used to measure hot moving energized, hard-to-reach objects instantly. Experimental results are given in Table 6 for generation of heat on disc surfaces. Disc surface temperatures increase with increasing braking time for different disc design configurations.

## 5. Experimental Validations

**Figure 7:**
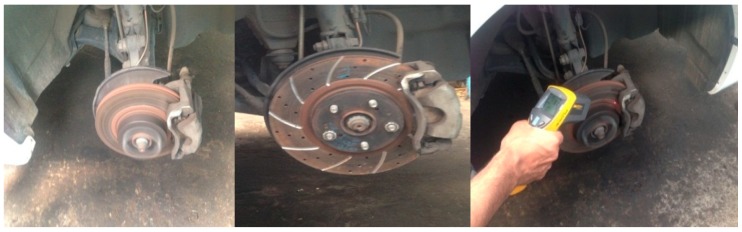
Temperature recording on modified brake disc surface with FLUKE IR thermometer.

The experimental has been conducted on disc brake with different radial grooves; Temperatures are recorded as shown in Figure 7 with Infra-Red Digital thermometer at different speeds. The heat flux were calculated are tabulated in Table 7 with the effect of radial grooves areas on disc brake.

## 6. Results and Discussions

**Figure 8:**
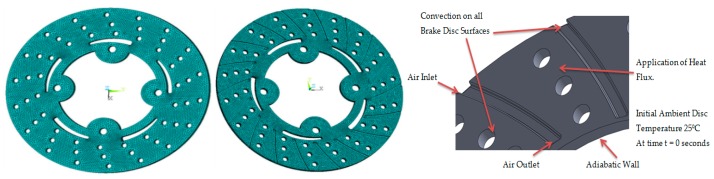
FE meshes model and radially grooved disc brake with boundary conditions.

Belhocine et al. [23,24,25] used FE software ANSYS for simulation of structural deformation of brake disc and observed induced stress depends on conditions such as speed, contact pressure and coefficient of friction. CAD model was created and FE analysis was performed using ANSYS 15. Tetrahedron Element type was selected for thermal analysis and it is a higher order four-node thermal element. Four-node elements have excellent compatible temperature shapes and are well suited to model curved boundaries and these elements are used for meshing grooved and un-grooved portion of disc brake shown in Figure 8, Transient thermal boundary conditions are introduced into thermal module of ANSYS 15.0, by choosing the first mode of simulation by defining material models and physical properties of materials [26,27]. The transient thermal simulation was carried out using simulation conditions tabulated in Table 8.

### 6.1. Nodal Temperatures and Contact Pressure

**Figure 9:**
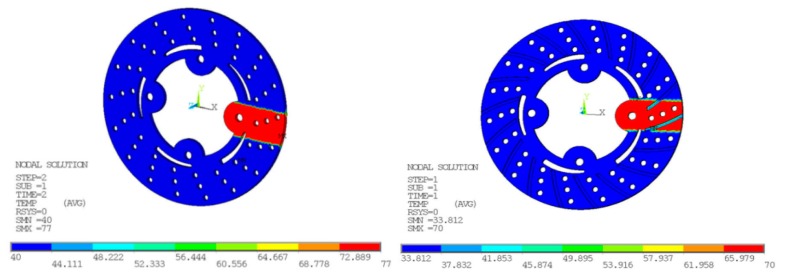
Nodal temperatures at braking pressure of 1 MPa, without and with grooves on disc surfaces.

**Figure 10:**
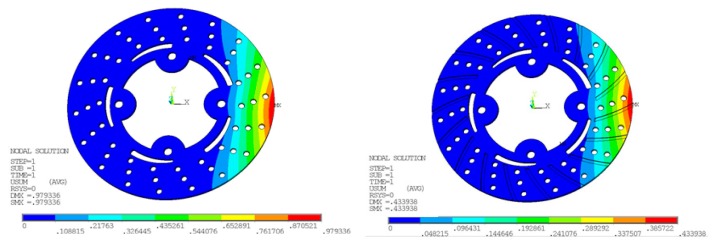
Thermal Deformation at Braking Pressure of 1 MPa, without and with grooves on disc surfaces.

**Figure 11:**
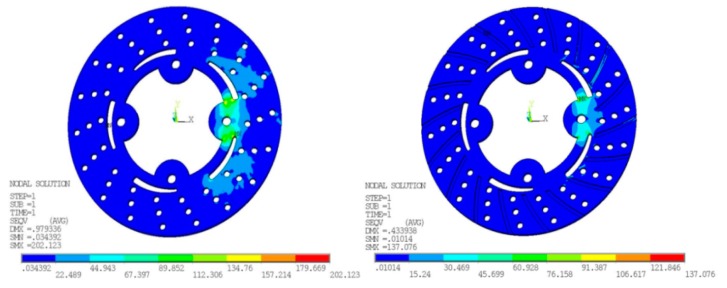
Von Mises and stress for conventional brake disc and radially grooved disc brake.

In Figure 9 and Figure 10, the temperature distribution of the disc brake is shown. Belhocine et al. [28,29], in their thermal studies on brake disc pad systems, tried to reduce thermal stresses by changing the design factors associated with the temperature distribution in disc brake and raises to squeal phenomenon. In comparison with experimental results, nodal temperatures obtained from FE simulation are in good agreement as surface temperatures Ts are reduced from 77 to 70.0 °C with and without radial grooves. The pressure distribution on disc surface area interface with brake pad taken at various times in FE simulation. For static deformation analysis, the pressure distribution scale varies from 0 to 3.0 MPa. The maximum contact pressure is located on edges of the brake disc and decreases from leading edge towards trailing edge due to friction. The maximum values of the thermal deformation vary from 979.336 to 433.938 µm with and without radial grooves, as shown in Figure 11. This value was obtained for speed of vehicle at 100 kph. The Comparative results of Static Deformation are given in Table 9. The same trend of the stress distribution was observed and highest stress areas are located in the same region with and without radial grooves. This pressure distribution is symmetrical compared with and without radial grooves. 

### 6.2. Von Mises and Stress

**Figure 12:**
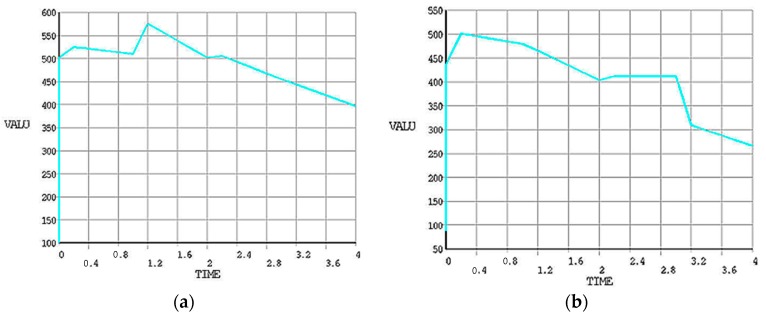
Transient FE analysis of (**a**) conventional and (**b**) grooved brake disc.

The maximum value recorded during this FE simulation for von Mises stresses at disc surface temperature 77 °C in the case without grooves is 202.123 N/mm^2^ while with grooves is 137.076 N/mm^2^. A significant reduction has been observed from ANSYS results. At higher speeds, the von Mises and stress are likely increased because of more frictional heat generated at the time of maximum braking time and pressure applied. The Transient finite element simulation gives variation of temperature distribution with respect to time as shown in Figure 12.

**Figure 13:**
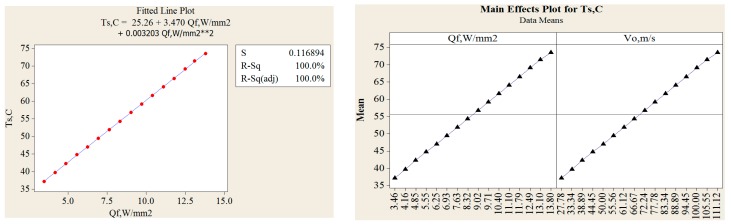
Fitted line plot for Temperature and heat flux and main effect plots for disc temperatures.

**Figure 14:**
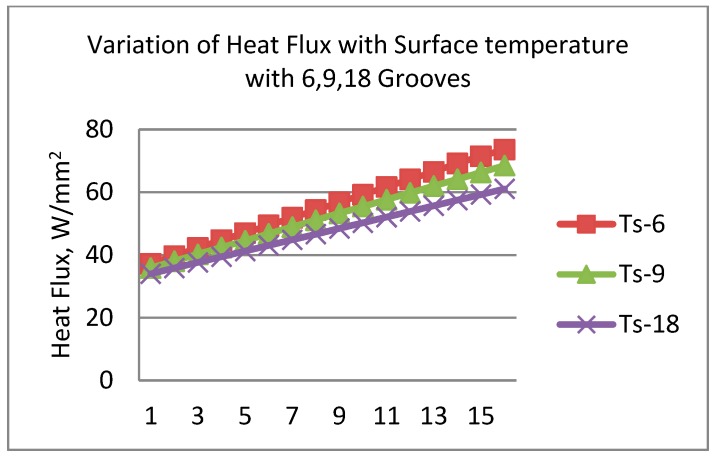
Variation of heat flux with surface temperature with 6, 9, and 18 Radial Grooves.

**Figure 15:**
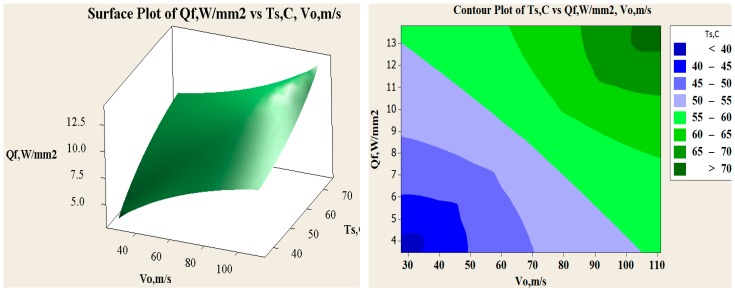
Variation of heat flux with different speed, surface temperatures and contour plots for surface temperature range at different heat flux.

In Figure 13, fitted line plot for heat flux and main effect plots for disc temperatures are shown. The different temperature profiles were observed with 6, 9 and 18 radial grooves but these temperatures are lower compared to disc brake without grooves, as shown in Figure 14. Estimation of heat flux is possible with these surface plots with respect to vehicle speeds. Transient FE analysis of conventional brake and grooved disc brake was carried out with simulated conditions given in Table 9. It has been observed that there is a decrease of temperature gradient after moving a certain distance after releasing of brake and with increasing speed of the vehicle as shown in Figure 15. The variation of disc surface temperatures was observed to increase with input heat flux, since, as the speed of the vehicle increases, the heat flux entering in o the disc also increases at the time of friction between two sliding surfaces and thereby sudden rise in temperature and fall at the braking time occurs. Main effect plots for disc surface temperatures represents information about amount of heat flux entered into the discs at respective speeds of the vehicle. Contour plot is typical graphical calculator that gives helpful information on design of disc brake under various thermal loading conditions. It is an additional tool to assess the surface temperatures and compare the nodal temperatures obtained through experimental and FE analysis. Experimentally determined temperatures with grooves and without grooves were 61.08 and 77.25 °C, respectively. It is in good agreement with 95% permissible confidence index. Maximum thermal stress is localized on corner of inner and outer edges of solid disc surfaces. variation in surface temperatures with different vehicle speed are presented in Table 10.

The maximum stress is located on inner surface side of holes. For the radially grooved disc configurations, maximum stresses occurred around holes that has been strengthened with more supports near the holes along the circumference of the disc. Hence, the specific regions where maximum stresses are observed have been strengthened to prevent potential crack and fatigue problems. The design modifications with radial grooves on disc surface could be one of the possible solutions for brake disc design for maximum heat dissipation, reduced thermal loading and less thermal fatigue. Maximum thermal stress is localized on the corner of the inner and outer edges of the solid disc surfaces. The maximum stress is located on inner surface and side holes in the disc.

## 7. Conclusions

In this research work, an attempt was made to study the effect of design modification on disc brake surface incorporated with radial grooves. FE Thermal Analysis was performed for analyzing thermal aspects with geometrical parameters influencing structural and thermal analysis of disc brakes without and with radial grooves. Surface modification on disc brake was achieved by additive manufacturing using DMLS process. Braking pressure, braking time and speed of the vehicle are influential parameters on thermal characterization of brake surface. From experimental results, it was concluded that 3D printed metals such as maraging steel can be used for longer life of disc brake with desired surface quality and it is suitable for high temperatures disc brake applications. Von Mises stresses were observed to be reduced in the case of grooved disc brake by 32% and also nodal temperature variation was around 10%. A transient thermal analysis was carried out using the direct time integration technique for the application of braking force due to friction for time duration of 4 s. The results obtained from this research work has revealed that, to enhance the maximum heat dissipation area on disc brake surface, radial grooves can be incorporated as they increase rate of heat transfer and reduce the disc surface temperature. Variation in heat flux for the 18 radial grooves are less when compare to 6 and 9 grooves. From the Transient temperature distribution, it has been observed that, for more braking time, the heat flux into the disc is increased and surface temperature also increases. It is recommended that using maraging steel disc brake material is safe based on the strength and rigidity criteria. All simulated values obtained from the finite element analysis are permitted values within the design tolerance and hence the brake disc design is safe based on comparative thermal analysis. The effective utilization of DMLS process can be further extended in designing and analyzing thermal deformation of brake disc pads developed through 3D printed materials and may be considered as future scope of present work.

## Figures and Tables

**Table 1:** Material Composition, wt % of maraging steel.

Metal	Wt %
Ni	17–19
Co	8.5–9.5
P/S	Max. 0.01
Mo	4.5–5.2
Ti	0.6–0.8
Al	0.052–0.15
C	Max. 0.03
Cr/Cu %	Max. 0.5
Fe	Balance

**Table 2:** 3D printed maraging steel properties.

Physical Properties	
1. Typical accuracy:	0–20 µm
2. Age hardening shrinkage:	0.08%
3. Smallest wall thickness:	0.3–0.4 mm
4. Relative density	100%
5. Specific density	8–8.1 g/cm^3^
6. Surface roughness Ra	4–6.5 µm
7. Surface roughness Rz	20 µm
Mechanical Properties	
1. Ultimate tensile strength:	2050 ± 100 MPa
2. Ultimate tensile strength (y)	1100 ± 100 MPa
3. Hardness	50–56 HRC
4. Tensile—Young’s Modulus	180,000 ± 20,000 MPa
Thermal Properties	
1. Thermal conductivity	20 ± 1 W/m °C
2. Specific heat capacity	450 ± 20 J/kg °C
3. Operating temperature:	400 °C

**Table 3:** Typical DSC specifications.

1. DSC Test Methods	ASTM E1269-05
2. Temperature Range	Ambient to 1100 °C.
3. Temperature Accuracy	±0.2 K
4. Temperature Precision	±0.02 K
5. Furnace-temperature Resolution	±0.00006 K
6. Heating Rate	0.02 to 300 K/min
7. Cooling Rate	0.02 to 50 K/min
8. Calorimetric Resolution	0.01 μ W
9. Measuring Environment	Nitrogen.
10. TG Measurement range	±400 mg
11. Scanning rate	0.01 °C to 150 °C/min
12. Sensitivity	0.2 μg.

**Table 4:** Properties of pad and disc.

Properties	Disc	Brake Pad
Young’s modulus (N/mm^2^)	180,000 ± 20,000 MPa	1500
Density (kg/m^3^)	8–8.1 g/cm^3^	2595
Poisson’s ratio	0.3	0.25
Thermal conductivity, W/m °C	20 ± 1 W/m °C	1.212
Ultimate tensile strength, N/mm^2^	2050 ± 100 MPa	-
Coefficient of friction	0.35	0.35
Specific heat (J/kg °C)	450 ± 20 J/kg °C	1465

**Table 5:** Input parameters and dimensions of brake discs.

External Brake disc Radius, mm	120
Internal Brake disc Radius, mm	60
Internal radius of pad, mm	60
External radius of pad, mm	120
Brake pad thickness, mm	12
Brake disc Thickness, mm	24
Brake disc Height, mm	49
Initial speed v, Km/h	30
Mass of Vehicle m, Kg.	1385
The cover angle of pad (in degrees), 20%	650
Deceleration ad, m/s^2^	8
Vent thickness, mm	6
Brake Disc Effective Radius, R effective, mm	100
Factor of Swept distribution of the disc, $\varepsilon_{p}$	0.5
Surface disc swept by the pad A cd, mm^2^	33,912
Contact Pressure P, MPa	1
Heat partition coefficient $\gamma_{p}$	0.95
Thermal Effusivity for Brake Pad.	2645.7
Thermal Effusivity for Brake Disc.	8971.3

**Table 6:** Calculation of heat flux at different influential parameters.

Time	$a_{d}$	∅	m	g	V0	z	A cd	$\varepsilon_{p}$	(1−∅)	$gmzV_{0}$	2 A cd ε_p_	Heat Flux
0	8	0.2	1385	9.8	33.34	0.8	0.033	0.5	0.8	369,407.2	0.03	4.925
1	8	0.2	1385	9.8	27.7	0.8	0.033	0.5	0.8	307,615.7	0.03	4.101
2	8	0.2	1385	9.8	22.2	0.8	0.033	0.5	0.8	246,269.8	0.03	3.283
3	8	0.2	1385	9.8	16.6	0.8	0.033	0.5	0.8	184,591.6	0.03	2.461
4	8	0.2	1385	9.8	11.1	0.8	0.033	0.5	0.8	123,134.9	0.03	1.641
5	8	0.2	1385	9.8	5.5	0.8	0.033	0.5	0.8	60,903.0	0.03	0.812

**Table 7:** Effect of radial grooves area on heat flux of disc brake.

	Disc Brake with 6-Grooves $A_{cd}=0.03637\;m^{2}$	Ts, °C	Disc Brake with 9-Grooves $A_{cd}=0.037602\;m^{2}$	Ts, °C	Disc Brake with 18-Grooves $A_{cd}=0.041292\;m^{2}$	Ts, °C
	Qo, W/mm^2^		Qo, W/mm^2^		Qo, W/mm^2^	
1.	3.383	36.61	3.272	35.87	2.979	34.02
2.	4.060	38.97	3.927	38.05	3.576	35.82
3.	4.736	41.27	4.581	40.22	4.171	37.62
4.	5.413	43.60	5.235	42.40	4.768	39.43
5.	6.089	45.92	5.889	44.57	5.363	41.23
6.	6.766	48.25	6.544	46.75	5.959	43.04
7.	7.443	50.58	7.199	48.93	6.556	44.84
8.	8.119	52.90	7.853	51.10	7.151	46.64
9.	8.797	55.23	8.509	53.28	7.749	48.45
10.	9.472	57.55	9.162	55.45	8.343	50.25
11.	10.14	59.88	9.817	57.63	8.939	52.06
12.	10.82	62.18	10.470	59.80	9.535	53.86
13.	11.50	64.53	11.125	61.98	10.131	55.67
14.	12.17	66.85	11.779	64.15	10.726	57.47
15.	12.85	69.16	12.433	66.33	11.322	59.27
16.	13.53	71.51	13.089	68.51	11.919	61.08

**Table 8:** Transient thermal simulation conditions.

3D-Printed Materials	Maraging Steel
Total time of simulation	45 s
Initial temperature of the disc	60 °C
Increment of initial time	0.25 s
Minimal Time Increment	0.5 s
Maximal Time Increment	0.125 s

**Table 9:** Comparison of Static Deformation results.

Parameters	Without Groove	With Groove
Nodal temperature	77 °C	70 °C
Thermal Deformation	0.979336 mm	0.433938 mm
Vonmises Stress	202.123 N/mm^2^	137.076 N/mm^2^

**Table 10:** Variation in temperatures with vehicle speed.

With Groove			Without Groove	
Vo, m/s	Experiment, Temp, (°C)	FEA, Temp, (°C)	Experiment, Temp, (°C)	FEA, Temp, (°C)
27.78	34.02	33.5	38.37	41.15
33.34	35.82	33.8	41.05	36.59
38.89	37.62	34.6	43.72	38.24
44.45	39.43	36.2	46.39	41.36
50.00	41.23	38.0	49.07	43.58
55.56	43.04	39.3	51.74	47.21
61.12	44.84	41.5	54.42	48.96
66.67	46.64	50.1	57.09	52.24
72.24	48.45	46.8	59.77	55.23
77.78	50.25	45.4	62.44	58.51
83.34	52.06	50.4	65.12	59.54
88.89	53.86	52.8	67.79	61.27
94.45	55.67	56.2	70.47	66.98
100.0	57.47	58.5	73.14	69.45
105.5	59.27	54.1	75.81	72.12
111.1	61.08	70.0	78.49	77.25

## Nomenclature

$M_{v}$	is total mass of vehicle
$V_{i}$	Initial speed of vehicle
$K_{e}$	Kinetic Energy
$\gamma_{p}$	Heat partition coefficient
$\xi_{\mathrm{ep}},{\;\xi }_{\mathrm{ed}}$	Thermal Effusivity of pad and Disc.
$A_{\mathrm{cp}},{\;A}_{\mathrm{cd}}\;$	Friction contact area of pad and Disc.
$Q_{\mathrm{Total}}$	Heat flux on contact area.
$Q_{\mathrm{disc}},{\;Q}_{\mathrm{Pad}}$	Heat flux into the disc and pad.
$Q_{f}$	Convective heat transfer
Ts	Surface temperature of brake disc.
Ta	Ambient air temperature in °C.
Ø	coverage sector of braking forces
Z	Braking Effectiveness.
g	Acceleration of gravity
$m_{a}$	Mass flow rate of air
$V_{\mathrm{avge}}$	Average air velocity
